# *Trypanosoma vivax* is the second leading cause of camel trypanosomosis in Sudan after *Trypanosoma evansi*

**DOI:** 10.1186/s13071-017-2117-5

**Published:** 2017-04-13

**Authors:** Ehab Mossaad, Bashir Salim, Keisuke Suganuma, Peter Musinguzi, Mohammed A. Hassan, E. A. Elamin, G. E. Mohammed, Amel O. Bakhiet, Xuenan Xuan, Rawan A. Satti, Noboru Inoue

**Affiliations:** 1 Department of Pathology, Parasitology and Microbiology, College of Veterinary Medicine, Sudan University of Science and Technology, P.O. Box 204, Khartoum, Sudan; 2 National Research Center for Protozoan Diseases, Obihiro University of Agriculture and Veterinary Medicine, Obihiro, Hokkaido 080-8555 Japan; 3 Faculty of Veterinary Medicine, University of Khartoum, P.O Box 32, Khartoum-North, Sudan; 4 Research Center for Global Agromedicine, Obihiro University of Agriculture and Veterinary Medicine, Obihiro, Hokkaido 080-8555 Japan; 5 Tsetse and Trypanosomosis Control Department, Central Veterinary Research Laboratory, Animal Resources Research Corporation, Ministry of Livestock, Fisheries and Rangelands, Khartoum, Sudan; 6 Obihiro University of Agriculture and Veterinary Medicine, Obihiro, Hokkaido 080-8555, Japan

**Keywords:** Dromedary camels, Sudan, Trypanosomosis, *Trypanosoma evansi*, *Trypanosoma vivax*

## Abstract

**Background:**

This study was conducted in response to recurring reports from eastern Sudan of camel trypanosomosis that can no longer be treated by currently available trypanocidal drugs. One hundred and eighty-nine blood samples were obtained from camels in different herds and local markets in the western part of Sudan, and a cross-sectional study was carried out between December 2015 and February 2016 to identify the causative agents and possible circulating genotypes.

**Results:**

The prevalence of trypanosomes detected using the conventional parasitological techniques of Giemsa-stained blood smears, wet blood smears and the microhematocrit centrifugation technique (MHCT) was 7% (13/189), 11% (21/189) and 19% (36/189), respectively. However, a multi-species KIN-PCR targeting the ITS region revealed that the prevalence of *Trypanosoma evansi* was 37% (70/189), while that of *T. vivax* was 25% (47/189). Consequently, we used a *T. evansi*-specific PCR (RoTat1.2 VSG gene) to analyse the KIN-PCR-positive samples and a *T. vivax-*specific PCR (Cathepsin L-like gene) to analyse all of the samples. The prevalence of *T. evansi* was 59% (41/70), while the prevalence of *T. vivax* was 31% (59/189). Mixed infections were detected in 18% (34/189) of the samples. These results were further confirmed by sequencing and a phylogenetic analysis of the complete internal transcribed spacer (ITS) region of *T. evansi* and the TviCatL gene of *T. vivax*.

**Conclusion:**

We conclude that *T. vivax* was newly introduced to the camel population and that *T. evansi* is no longer the single cause of camel trypanosomosis in Sudan. The presence of *T. vivax* in camels detected in this study is a challenge in the choice of diagnostic approaches, particularly serology, and PCRs. However, an analysis of drug resistance should be performed, and the genotypic variation should be verified. To our knowledge, this is the first molecular study on *T. vivax* and mixed-infection with *T. vivax* and *T. evansi* in Sudanese camels.

**Electronic supplementary material:**

The online version of this article (doi:10.1186/s13071-017-2117-5) contains supplementary material, which is available to authorized users.

## Background

With 4,623,000 camels (*Camelus dromedaries*), Sudan has the second largest camel population in the world after Somalia (FAO and the Annual Report of the Ministry of Animal Resources, Fisheries and Ranges, 2010). These dromedaries, which are sustainably used in arid and hostile environments, provide food and transport for millions of people in the marginal agricultural areas of Sudan and throughout the world. Trypanosomosis, which is caused by *Trypanosoma evansi*, a parasite that infects livestock and a potential human pathogen, is a major threat to these valuable animals [1, 2]. *Trypanosoma evansi* has multiple and complex means of transmission depending on the host and the geographical area. Biting and sucking insects transmit the parasite mechanically and the transmission can be vertical, horizontal, iatrogenic, or per-oral, each of which has different epidemiological significance depending on the season, location, and host species [3]. In Sudan, cattle, sheep and goats undergo protracted infection in which they may play the role of a reservoir host [4]. In addition, co-herding may increase the possibility of infection with *T. evansi* and other trypanosomes [5].

Clinical signs and pathological lesions are unreliable for definitively diagnosing these infections in camels [6]. Parasitological examinations suffer from limited sensitivity [7, 8] and serological tests cannot distinguish between past and current infections as the antibodies persist in the circulation [8].

In Sudan, the disease known as “Guffar” is a serious protozoan disease of camels. The disease was first reported in the country in 1904 [5, 9]. Since then, various studies have used parasitological and serological tests to investigate the epidemiology of camel trypanosomosis in different parts of the country [4, 10–13]. Very recently, a few reports have described the performance of molecular studies [14–16].

To date, all of the reports on camel trypanosomosis in Sudan have indicated that *T. evansi* is the sole pathogenic trypanosome infecting camels [12, 13, 15, 16]; no other *Trypanosoma* spp. have been documented thus far. To this end, we conducted the present study in response to recurring reports from eastern Sudan of camel trypanosomoses that no longer respond to the currently available trypanocidal drugs. We aimed to obtain information on the current prevalence of the disease as well as identify the causative agents and possible genotypes circulating in the area.

## Methods

### Study area and sample collection

**Fig. 1:**
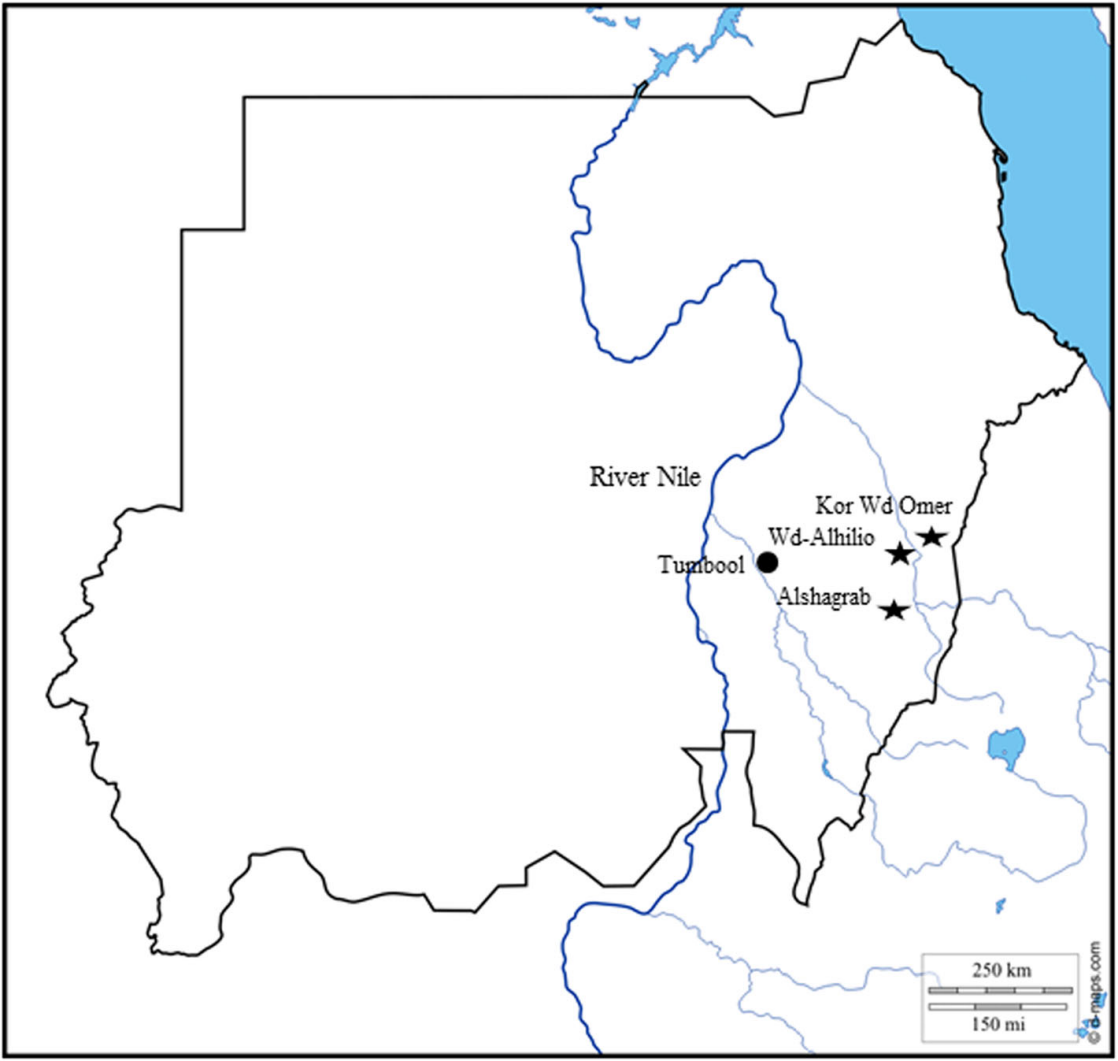
A map of Sudan: The locations of the sampling areas from different herds are shown with *black stars*. The *black dot* indicates Tumbool slaughterhouse. Source: http://www.d-maps.com/carte.php?num_car=1310&lang=en. With some modifications

Samples were obtained from 189 camels from three herds in Wd-Alhlio, Alshagrab and Khor Wd-Omer, which are located around El-Showak in Kassala state (*n* = 148), and from Tumbool market (at a camel slaughterhouse) in El-Gazira state (*n* = 41) where camels are brought from western Sudan, across the natural barrier of the River Nile (see map in Fig. 1). After obtaining the consent of the camels’ owners, 3 ml of blood was drawn from the jugular vein into vacutainer tubes with EDTA (Terumo, Tokyo, Japan). The samples were labelled with a unique code and were placed in a cool box at 4 °C until they were transported to the laboratory.

### Conventional parasitological examinations

All the blood samples were examined *in situ* for the presence of trypanosome species using wet blood films [17], the microhaematocrit centrifugation technique (MHCT), the packed cell volume (PCV) technique described by Mamoudou et al. [18] and 10% Giemsa thin blood smears, according to the methods of Murray et al. [17], and the OIE guidelines [19].

### DNA extraction and the PCR-based identification of the trypanosome species

Genomic DNA was extracted from all blood samples using a QIAamp DNA Blood Mini Kit (Qiagen, Hilden, Germany), in accordance with the manufacturer's instructions, and four different PCR reactions were employed to detect and identify trypanosome DNA in camels. These included: (i) a KIN-multi species PCR, which amplifies the internal transcribed spacer 1 (ITS1) and allows for the simultaneous detection of three major trypanosome species (*T. evansi*, *T. congolense* and *T. vivax*) [20]; (ii) the RoTat 1.2 VSG PCR (*T. evansi* type A-specific), which specifically amplifies the RoTat1.2 VSG gene encoding the variable surface glycoprotein (VSG) of *T. evansi* [21]; (iii) the TviCatL PCR, which amplifies the Cathepsin L-like gene, which is highly conserved among *T. vivax* isolates [22]; and (iv) the internal transcribed spacers (ITS), which are versatile genetic markers that are used for phylogenetic analyses, the evaluation of the evolutionary process and for the determination of taxonomic identities [23, 24]. All the primer sequences used in the PCRs are listed in Table 1. Trypanosomes were detected using single-step PCR methods with a total reaction volume of 10 μl, which included 1 μl of 10× reaction buffer, 0.3 μl of 50 mM magnesium chloride, 1 μl of 250 μM dNTPs, 0.1 μl of *Taq* DNA polymerase (Invitrogen™, Thermo Fisher Scientific Inc., MA, USA), 1 μl each of 10 mM forward and reverse primers, 4.6 μl of double-distilled water and 1 μl of the DNA sample, which was added to the individual PCR mixtures. PCRs were conducted on a Veriti™ Thermal Cycler (Thermo Fisher Scientific). The PCR conditions that were used for the KIN-PCR and the TviCatL-PCR were previously described by Laohasinnarong et al. [25], while the conditions of the RoTat 1.2 VSG-PCR were described by Urakawa et al. [21]. The PCR products were electrophoresed in 2% agarose gels, stained with ethidium bromide, and visualised under ultraviolet light.

**Table 1:** The primers used in the present study

Parasite	Method	Primer	Sequence (5′–3′)	Target gene	Fragment size (bp)	Reference
*T. evansi*	KIN-PCR	Kin1	GCGTTCAAAGATTGGGCAAT	ITS1	540	Desquesnes et al. [20]
		Kin2	CGCCCGAAAGTTCACC			
*T. evansi*	RoTat 1.2-PCR	ILO7957	GCCACCACGGCGAAAGAC	RoTat 1.2 VSG	488	Urakawa et al. [21]
		ILO8091	TAATCAGTGTGGTGTGC			
*T. evansi*	ITS-PCR	IR1	GCTGTAGGTGAACTTGCAGCAGCTGGATCATT	ITS	1100	Da Silva et al. [23]
		IR2	GCGGGTAGTCCTGCCAAACACTCAGGTCTG			
*T. vivax*	KIN-PCR	Kin1	GCGTTCAAAGATTGGGCAAT	ITS1	300	Desquesnes et al. [20]
		Kin2	CGCCCGAAAGTTCACC			
*T. vivax*	TviCatL-PCR	DTO 155	TTAAAGCTTCCACGAGTTCTTGATGATCCAGTA	Cathepsin L-like	200	Cortez et al. [22]
		TviCatL1	GCCATCGCCAAGTACCTCGCCGA			

### Cloning and DNA sequencing of *T. evansi* ITS and *T. vivax* TviCatL genes

The PCR for *T. evansi* ITS, was performed in 20 μl of reaction mixture containing 4 μl of 5× Phusion® HF Buffer (1.5 mM MgCl_2_ was included in the final concentration), 1.6 μl of 200 μM dNTPs, 1 μl each of 1 μM IR1 and IR2 as a final concentration, 0.2 μl of Phusion® DNA polymerase (BioLabs, New England, USA) and 10.2 μl of sterile deionized distilled water. The amplification of ITS was performed for 40 cycles; each cycle consisted of denaturation at 98 °C for 10 s, annealing at 62 °C for 30 s and extension at 72 °C for 1 min. The entire ITS amplicon was gel-extracted using a QIAamp gel extraction kit (Qiagen), cloned and transformed to chemically-competent *Escherichia coli* (One Shot® Mach1™; ThermoFisher Scientific) using the TOPO® cloning procedure in accordance with the manufacturer’s instructions. After checking the cloned products by colony PCR, 6 clones of ITS were selected for further plasmid DNA purification using a QIAamp Spin Miniprep Kit (Qiagen). Approximately 300 ng/μl of pure plasmid DNA was used for sequencing using a Big Dye Terminator kit (Applied Biosystems, Austin, USA). The cycle sequencing procedure consisted of 30 cycles of denaturation at 96 °C for 1 min, annealing at 50 °C for 5 s and extension at 60 °C for 2 min. The PCR product was ethanol-precipitated and dissolved in 20 μl of Hi-Di formamide solution before DNA sequencing. The gene sequence was analysed using an ABI Prism 3100 Genetic Analyzer (Applied Biosystems, Carlsbad, CA, USA). Both forward and reverse primers were used to construct a continuous sequence of inserted DNA. In contrast, the TviCatL-PCR products of *T. vivax* were excised from the gel and purified using a QIAamp gel extraction kit (Qiagen), and were subsequently subjected to direct sequencing using a Big Dye Terminator kit, as mentioned above.

### Sequence analysis and phylogenetic analysis

The ITS region of *T. evansi* (ITS1 + 5.8S + ITS2 rDNA, ~1100 bp) and the TviCatL-PCR sequences (~200 bp) of *T. vivax* were each edited manually to correct possible base calling errors using the BioEdit 7.0 software program [26] and were subsequently joined to reconstruct a 950 bp fragment of the ITS gene and a 160 bp fragment of the TviCatL gene. The consensus sequences were aligned with sequences that were publicly available in the GenBank database using the Clustal X 2.1 software program [27].

Phylogenetic trees were constructed using the neighbour-joining method implemented in the Mega software program (version 6.0) [28]. The best substitution models, as determined by the model test algorithm [29] implemented in the Mega software program were T92 + G and K2 + I for ITS-*T. evansi*. All sequences generated in this study were deposited in the GenBank database under the accession numbers (LC199490, LC199491, LC198227, LC198229–LC198233).

### Statistical analysis

A chi-squared test to investigate the differences in the prevalence of trypanosome infections in the two study areas was performed using the GraphPad Prism software program (GraphPad Software Inc., CA, USA). Cohen’s kappa coefficient was calculated using VassarStats: Website for Statistical Computation (http://www.vassarstats.net/kappa.html); the results were interpreted according to a previously described method [30]. The associations between the hematocrit values in the infected and non-infected animals (with each of the trypanosomes), as determined by different diagnostic tests, were analysed using Student's *t*-test. *P-*values were determined using the GraphPad Prism software program (GraphPad Software Inc., CA, USA). *P-*values of < 0.05 were considered to indicate statistical significance.

## Results

### Prevalence of *T. evansi* in camels

**Fig. 2:**
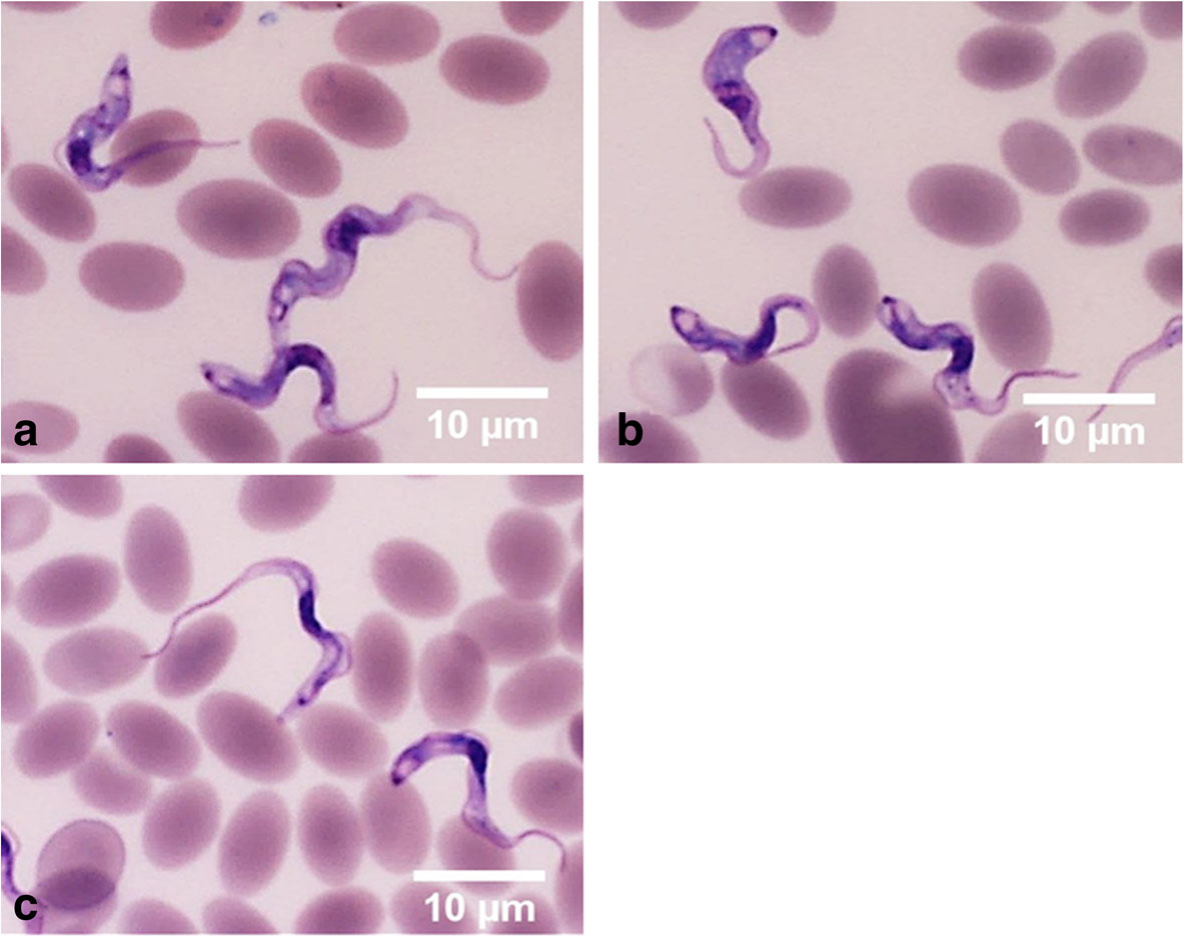
Light micrographs of Giemsa-stained blood smears from camel samples. **a** *Trypanosoma evansi* with a small subterminal kinetoplast at the pointed posterior end, a long free flagellum and a well-developed undulating membrane. **b** *Trypanosoma vivax* with a long free flagellum, an inconspicuous undulating membrane, a rounded posterior end and a large terminal kinetoplast. **c** Mixed infection. *Scale-bars*: 10 μm

Direct wet blood films showed the presence of trypanosomes in 11% (21/189) of the camel samples, 19 of which showed the typical movement of *T. evansi*. Two samples showed trypanosomes that moved forward quickly, the characteristic movement pattern of *T. vivax* [31] (Table 2). However, the concentration of trypanosomes, as measured by MHCT, revealed trypanosome-positivity in 19% (36/189) of the samples (Table 2). Additionally, Giemsa-stained thin blood films revealed trypanosome-positivity in 7% (13/189) of the samples (Table 2). In accordance with the guidelines of Connor & Van den Bossche [32], which describe the morphology of different trypanosomes in thin blood smears based microscopic observation, *T. evansi* infection was identified in 10 samples*, T. vivax* was identified in 2 samples, and mixed infection was identified in 1 sample (Fig. 2a-c). Nevertheless, the prevalence estimated by the three tests did not differ to a statistically significant extent.

**Table 2:** The prevalence of *T. evansi*, *T. vivax* and mixed infection in camels with different diagnostic tests

Area	Giemsa-stained blood smears^a^	Wet blood film^b^	MHCT	KIN-PCR *T. evansi*	RoTat 1.2 VSG-PCR^c^	KIN-PCR *T. vivax*	TviCatL-PCR	Mixed infection^d^
East Nile	9% (13/148)	14% (20/148)	22% (33/148)	36% (54/148)	59% (32/54)	25% (37/148)	33% (49/148)	19% (28/148)
West Nile	0% ( 0.0/41)	3% (1/41)	7% (3/41)	39% (16/41)	56%(9/16)	24% (10/41)	24% (10/41)	15% (6/41)
Total	7% (13/189)	11% (21/189)	19% (36/189)	37% (70/189)	59% (41/70)	25% (47/189)	31% (59/189)	18% (34/189)

^a^Giemsa-stained blood smears: 10 samples showed typical *T. evansi* morphology, 2 samples showed typical *T. vivax* morphology and 1 sample showed mixed infection

^b^Wet blood film: 19 samples showed a typical *T. evansi* movement pattern while 2 samples showed a typical *T. vivax* movement pattern

^c^A RoTat 1.2 VSG-PCR was performed on KIN-PCR-positive samples (70 samples)

^d^Mixed infection was defined by KIN-PCR-positivity for *T. evansi* and TviCatL-PCR-positivity for *T. vivax*

On the other hand, the KIN-PCR, which targets the ITS1 region, which is conserved in all African trypanosomes, revealed that 37% (70/189) of the samples contained a 540 bp PCR product, indicating the amplification of a *Trypanozoon* that corresponded to *T. evansi* (the Tsetse free zone) (Additional file 1: Figure S1). This result was later confirmed. The prevalence in the eastern Nile area was 36% (54/148), while that in the western Nile area was 39% (16/41) (Table 2). No statistical significant difference in the prevalence of trypanosome infection was observed between the two study areas. After that, all of the KIN-PCR-positive samples were further subjected to the *T. evansi* RoTat1.2 VSG species-specific PCR. As a result, a 488 bp PCR product was detected in 59% (41/70) of the samples (Additional file 2: Figure S2). The prevalence in the eastern Nile area was 59% (32/54) while that in the western Nile area was 56% (9/16) (Table 2).

**Fig. 3:**
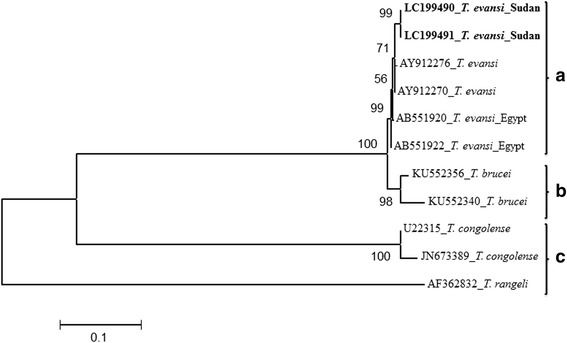
Confirmation of *T. evansi* identified in this study by the neighbour-joining phylogenetic tree that shown well clustering with reference sequences of *T. evansi*. The relationship was determined using the ITS of rRNA gene sequences by neighbour-joining with 1000 bootstraps. *T. evansi* identified in this study were depicted in bold letters. Trypanosomes sequences from GenBank were shown both by their accession numbers and parasites names. Scale bar used was nucleotide substitutions per site

Confirmation of the 540 bp PCR product of *Trypanozoon* as *T. evansi* was achieved by arbitrary selection, cloning and the complete sequencing of the ITS (ITS1 + 5.8S + ITS2 rDNA) of two positive samples (Additional file 3: Figure S3). The sequence similarity and the phylogenetic analysis confirmed the entity as *T. evansi* by grouping it with other *T. evansi* strains, in particular, the strains from Egypt, which shares a border with Sudan (Fig. 3). The two sequences were deposited in the GenBank database under the accession numbers LC199490 and LC199491.

### Prevalence of *T. vivax* in camels

Two PCRs were performed to detect *T. vivax* in the present study. First, the KIN-PCR that was used to detect *T. evansi* (540 bp = *Trypanozoon*) generated a 300 bp amplicon, indicating the presence of *T. vivax* in 25% (47/189) of the samples. The prevalence in the samples from the eastern Nile area was 25% (37/148), while that in the samples from the western Nile area was 24% (10/41) (Additional file 1: Figure S1; Table 2). Secondly, the TviCatL-species-specific PCR generated a 200 bp amplicon of the *T. vivax* CatL-like gene. This revealed a higher prevalence of 31% (59/189). The prevalence in the eastern Nile area was 33% (49/148), while that in the western Nile area was 24% (10/41) (Additional file 4: Figure S4; Table 2). The prevalence detected by the two PCRs did not differ to a statistically significant extent. We further confirmed the identity of the 200 bp-positive samples as *T. vivax* by the direct sequencing of 10 selected samples, as well a Basic Local Alignment Search Tool (BLAST) analysis. Six of these sequences were deposited in GenBank under the accession numbers LC198227, LC198229–LC198233.

### Mixed infection with *T. evansi* and *T. vivax* in camels

The overall prevalence of mixed infection with the two parasites, detected using both the KIN-PCR and the TviCatL-species-specific PCR, was 18% (34/189). The prevalence in the eastern Nile area was 19% (28/148), while that in the western Nile area was 15% (6/41) (Table 2). The prevalence of mixed infection in the two areas did not differ to a statistically significant extent.

### The association between a reduced PCV and anemia

**Fig. 4:**
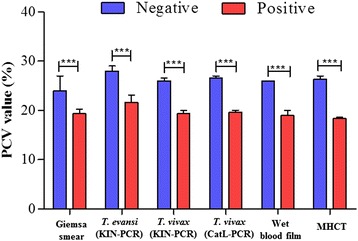
Comparison of PCV values between trypanosome infected and non-infected camels detected with different diagnostic tests. Values are mean ± SD. The significant difference by Student's *t*-test (***P < 0.0001)

Because anemia is one of the consequences of trypanosome infections [33] and low a PCV is an indicator of anemia in animals, the mean PCVs in both *T. evansi*- and *T. vivax-*positive animals were significantly low in comparison to their test-negative counterparts (Student's *t*-test, *df* = 10, *P* < 0.0001) (Fig. 4).

### Agreement between the diagnostic tests

Sixteen and 13 of the 70 KIN-PCR-positive samples were also found to be positive using wet blood films and thin blood films, respectively. Regarding the negative samples, 3 of the 119 KIN-PCR-negative samples were found to be positive by wet blood films; none were found to be positive using thin blood films (Table 3). There was a fair agreement between the KIN-PCR results and the results of both wet blood films (κ = 0.2334) and thin blood films (κ = 0.3057).

**Table 3:** The detection of *T. evansi* in camels. The KIN-PCR results were cross-tabulated with those of wet blood films and thin blood films

	KIN-PCR		Wet blood film		Thin blood film	
			(+)	(−)	(+)	(−)
*T. evansi*	(+)	70	16	54	13	57
	(−)	119	3	116	0	119

For *T. vivax*, 40 of the 47 KIN-PCR-positive samples were also found to be positive by the TviCatL-PCR, while 19 of the 142 KIN-PCR-negative samples were found to be positive by the TviCatL-PCR (Table 4). There was substantial agreement between the KIN-PCR and the TviCatL-PCR results (κ = 0.6608).

**Table 4:** The detection *of T. vivax* in camels

	KIN-PCR		TviCatL-PC	
			(+)	(−)
*T. vivax*	(+)	47	40	7
	(−)	142	19	123

The KIN-PCR results were cross-tabulated with the TviCalL-PCR results

## Discussion

Ever since the first report of camel trypanosomosis in 1904 [9] and until 2011 [15], *T. evansi* has been the only reported causative agent of the disease. In the present study, we documented, for the first time, that *T. vivax* was highly prevalent in Sudan. The parasite was detected in 31% (59/189) of the camels in the present study. This finding is supported by the detection of the parasite by various parasitological tests and confirmed by the detection and identification of its DNA by PCRs and sequencing analysis. Thus, we argue that *T. vivax* is a newly emerging cause of the disease in the country. This argument is strongly supported by the study of Salim et al. [15] who only detected *T. evansi* in the screening of 687 camels from geographically different areas of the country using molecular detection methods. This may possibly be attributed to various causes, including, but not limited to, climate change, limited access to veterinary services and follow-up, and the displacement of people and their animals from the Blue Nile State in the southeastern part of the country (a Tsetse-endemic area) and from the southern Kordofan State to the northern part of the country, due to a conflict that has taken place since 2011 [34].

The detection of trypanosomes in 7% (13/189) of the camel samples using Giemsa-stained blood smears is compatible with the prevalence reported by Ali et al. [16], which was 6% in Sudan and 6.5% in Ethiopia [35, 36]. However, it was higher than the prevalence reported in the previous year, which was 2% in Ethiopia [37], 2.3% in Kenya [38] and 2.5% in Nigeria [39]. This could be due to the differences in animal husbandry and management practices, seasonal effects, or the study design. It is worth mentioning that all of the Giemsa-positive animals were from the eastern Nile area. No animals from the western Nile area were found to be positive. This might be because the animals from the western Nile area (*n* = 41) were apparently healthy and well fed since they had been prepared to slaughter for human consumption, which resulted in their parasitemia being lower than the detectable level. This could also be why the other parasitological technique showed the same pattern of detection within the two areas.

The prevalence of 19% (36/189) obtained using MHCT was higher than that in a previous study which reported that the prevalence in eastern Sudan was 5.4% [12]. The prevalence detected by wet blood films was 11% (21/189). This was comparable to a previous study, which reported that the prevalence was 14.1% [13]. The high parasitological prevalence might also be due to the spread of drug resistance, which was reported in previous studies [13, 40].

General, Giemsa-stained blood smears and wet blood films have been found to be useful for recognising and differentiating *T. evansi* and *T. vivax* based on their characteristic morphology and movement patterns. However, it is difficult to differentiate between the parasites using MHCT because of the massive movement of the parasites within the small area of the buffy coat within the capillary tube.

The detection of *T. evansi* and *T. vivax* in the sampled camels using less-sensitive parasitological examination methods confirmed the results and provided a guide to the molecular prevalence of the parasites. This proved that molecular detection outperformed conventional parasitological techniques by identifying parasites at the species level with 100% credibility and revealed high prevalence and mixed infection with higher resolution (see Results). For instance, the PCRs performed using universal primers to simultaneously detect and differentiate the *T. brucei* group, *T. congolense* and *T. vivax* by amplifying ITS1 using the so-called KIN-multi-species PCR procedure [20] revealed that 37% (70/189) of the samples were positive for *Trypanozoon*. This rate was significantly higher than the prevalence detected using Giemsa-stained thin blood smears (7%; 13/189), wet blood films (11%; 21/189) and MHCT (19%; 36/189). The 70 *Trypanozoon-*positive samples were further confirmed to be *T. evansi-*positive by a RoTat 1.2 VSG-species specific PCR and by a sequencing analysis of the ITS region of some of the KIN-PCR-positive samples. However, while the RoTat 1.2 VSG-species-specific PCR detected 41 (59%) positive samples (among the 70 *Trypanozoon-*positive samples), the KIN-PCR detected an additional 29 positive samples. This could be attributed to the limitation of the RoTat 1.2 VSG-PCR in detecting RoTat 1.2 VSG-negative *T. evansi*, which has been previously reported in Sudanese camels [15]. Thus, these 29 negative samples were regarded as *T. evansi* type B [15, 41].

Similarly, KIN-PCR simultaneously detected *T. vivax* in 25% (47/189) of the samples*.* The TviCatL-species-specific further confirmed the result and revealed a higher prevalence of 31%. Although the TviCatL-PCR has been shown to have greater sensitivity than the KIN-PCR in detecting *T. vivax*, we observed substantial agreement between the two tests (κ = 0.6608). Conversely, the KIN-PCR has been reported to have limited ability in the detection of *T. vivax* [20, 25, 42], because it is a very diverse parasite, with three main groups of isolates: (i) East African; (ii) West African; and (iii) South American isolates [22]. Thus, *T. vivax* may be difficult to detect using a single PCR. However, in the present study, comparable numbers of samples from camels in Sudan were found to be *T. vivax-*positive by the KIN and TviCatL PCRs. Thus, additional research should be conducted to verify the possible diversity of *T. vivax* in Sudan. We have also reported the mixed infection between *T. evansi* and *T. vivax* in 18% (34/189) which is higher than that reported by Birhanu et al. in Ethiopia [43].

The overall prevalence of *T. evansi* and *T. vivax,* as detected using parasitological techniques (Giemsa thin blood smears, wet blood smears and MHCT), was significantly lower in comparison to the prevalence detected by molecular tests (KIN-PCR for *T. evansi* and KIN-PCR and TviCatL-PCR for *T. vivax*) (*P* < 0.05). In all the infected animals, the PCVs were lower than the PCVs in non-infected animals. This was also clearly demonstrated in *T. vivax*-infected camels. Similar results were obtained in Ethiopia by Birhanu et al. [43]. In some of the individual camels infected with *T. evansi* or *T. vivax*, the PCV was as low as 9%, while PCVs of as low as 24% were measured in some apparently healthy animals that were in good physical condition. This indicates that caution must be exercised when measuring the PCV to avoid a misdiagnosis.

The paradigm that camel trypanosomosis is caused by a single parasite species (*T. evansi*) is no longer valid after our detection of *T. vivax,* as a second causative agent of the disease. Consequently, these findings should alert veterinary authorities of the need to safeguard approximately 5 million camels in Sudan. Ultimately, the disease caused by *T. vivax* has a different clinical presentation with high pathogenicity [44], which might cause higher morbidity and mortality rates alongside *T. evansi*, which on some occasions is also known to be highly pathogenic in camels. It is worth mentioning that *T. vivax* infection is also transmitted mechanically, as occurs in the case of *T. evansi*, by several *Tabanids* and a range of biting flies [45, 46]. This makes the transmission and the spread of infection possible in a wide range of camel populations in Sudan. In the same context, *T. vivax* was reported to have spread widely, as far as North Sudan, which is located hundreds of kilometres from the tsetse belt, a region that includes camel breeding areas [47].

In Ethiopia, which shares a border with eastern Sudan, Fikru et al. [37] recently confirmed *T. vivax* in camels. This suggests that the emergence of *T. vivax* infection will be a future regional challenge in at least two countries, with a total camel population of over 6 million. Similarly, Mbaya et al. [48] reported *T. vivax* in camels in Nigeria, which is in western Africa.

## Conclusions

We documented, for the first time, the high prevalence of *T. vivax* in camels from eastern Sudan, which, until five years previously, was reported to be free of the parasite. The presence of *T. vivax* in camels detected in this study is a challenge in the choice of diagnostic approaches, particularly serology and PCRs. Furthermore, the findings should alert veterinary authorities of the need to look carefully for an effective combination that can be used in the treatment of this devastating disease, which has been newly emerged in the area. The finding that camel trypanosomosis caused by *T. vivax* or mixed infection with *T. evansi* is highly prevalent in the country suggests the need for stringent control policies and the establishment of measures to help prevent the spread of the parasites. We also recommend that the disease status is updated throughout the country, as we anticipate that there will have been a marked change in the situation.

## Additional files

Additional file 1: Figure S1. Agarose gel electrophoresis (2%) with ethidium bromide staining of field isolates of *T. evansi* and *T. vivax.* The DNA was amplified with a KIN-PCR (kin1 and kin2 primers). Lane M: 100-bp marker; Lane 1: negative control; Lane 2: positive control (*T. evansi*); Lanes 3–7 and 9–11: positives for *T. evansi* (540 bp); Lanes 8 and 12: negative samples. *T. vivax*, Lanes 4, 5, 7, 8 and 11: positives for *T. vivax* (300 bp); Lanes 3, 6, 9 and 12: negative samples. Mixed infection, Lanes 4, 5, 7 and 11. The extra-bands were non-specific. (TIF 110 kb)

Additional file 2: Figure S2. Agarose gel electrophoresis (2%) with ethidium bromide staining of field isolates of *T. evansi* (obtained in the field). The DNA was amplified with a RoTat 1.2 VSG-PCR. Lane M: 100-bp marker; Lane 1: negative control; Lane 2: positive control (*T. evansi*); Lanes 4–8 and 10–11: positives for *T. evansi* (488 bp); Lanes 3 and 9: negative samples. (TIF 86 kb)

Additional file 3: Figure S3. Agarose gel electrophoresis (1.5%) with ethidium bromide staining of field isolates of *T. evansi.* DNA was amplified with an ITS-PCR (IR1 and IR2 primers). Lane M: 100-bp marker; Lane 1: negative control; Lane 2: positive control (*T. evansi*); Lanes 3–6: positives for *T. evansi* (1.1 kbp); Lanes 7 and 8: negative samples. The extra-bands were non-specific. (TIF 144 kb)

Additional file 4: Figure S4. Agarose gel electrophoresis (2%) with ethidium bromide staining of field isolates of *T. vivax.* DNA was amplified with a TviCatL-PCR (DTO155 and TviCatL1 primers). Lane M: 100-bp marker; Lane 1: negative control; Lanes 2–7 and 9–15: positives for *T. vivax* (200 bp); Lane 16: positive control (*T. vivax*); Lane 8: negative sample. The extra-bands were non-specific. (TIF 122 kb)

## Abbreviations

BLAST: Basic local alignment search tool
ITS: Internal transcribed spacer
MHCT: Microhematocrit centrifugation technique
OIE: Office international des epizooties
PCV: Packed cell volume
κ: Kappa value
