# Supplementary figures and images for: Trypanosoma vivax is the second leading cause of camel trypanosomosis in Sudan after Trypanosoma evansi
Source: Parasit Vectors. 2017 Apr 13;10:176. doi: 10.1186/s13071-017-2117-5 (PMC5390396; doi:10.1186/s13071-017-2117-5)

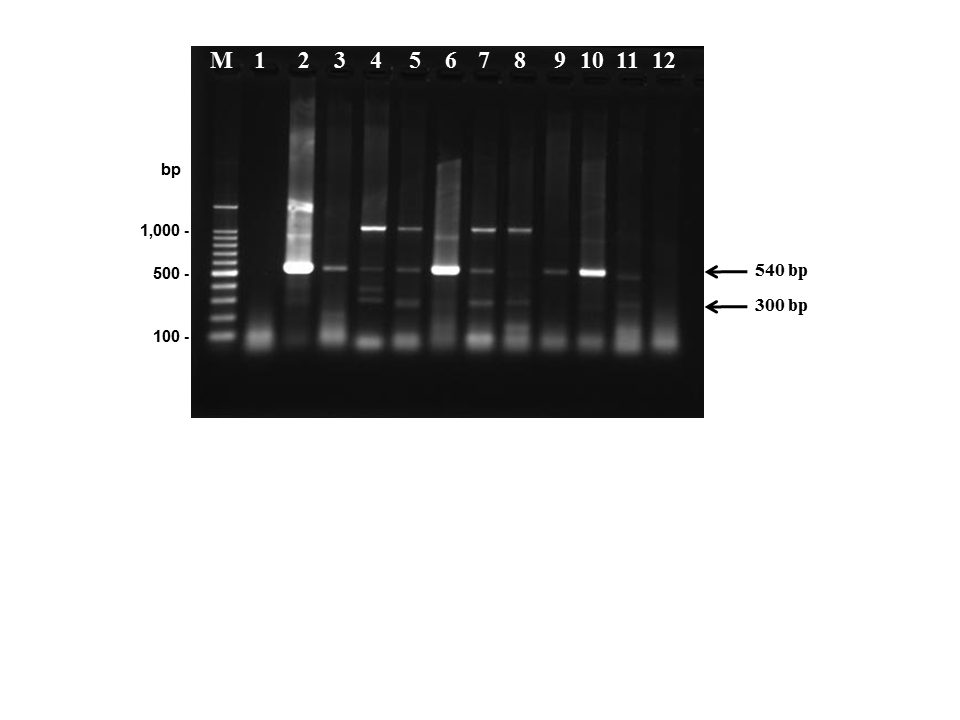

Supplement: Supplementary file 1 — Agarose gel electrophoresis (2%) with ethidium bromide staining of field isolates of T. evansi and T. vivax. The DNA was amplified with a KIN-PCR (kin1 and kin2 primers). Lane M: 100-bp marker; Lane 1: negative control; Lane 2: positive control (T. evansi); Lanes 3–7 and 9–11: positives for T. evansi (540 bp); Lanes 8 and 12: negative samples. T. vivax, Lanes 4, 5, 7, 8 and 11: positives for T. vivax (300 bp); Lanes 3, 6, 9 and 12: negative samples. Mixed infection, Lanes 4, 5, 7 and 11. The extra-bands were non-specific. (TIF 110 kb) [file 13071_2017_2117_MOESM1_ESM.tif]

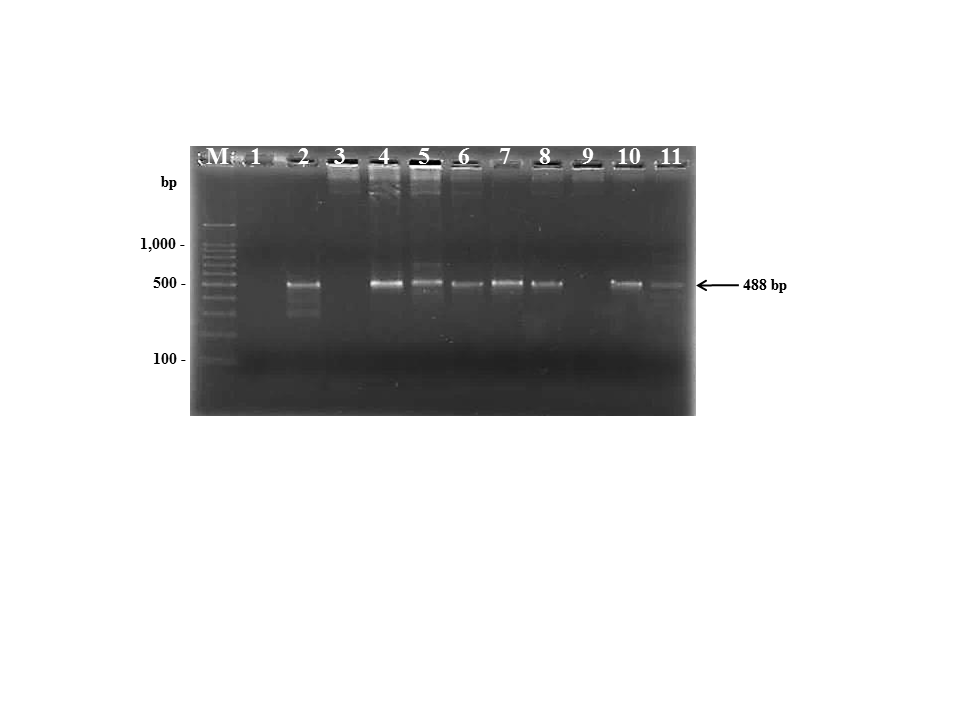

Supplement: Supplementary file 2 — Agarose gel electrophoresis (2%) with ethidium bromide staining of field isolates of T. evansi (obtained in the field). The DNA was amplified with a RoTat 1.2 VSG-PCR. Lane M: 100-bp marker; Lane 1: negative control; Lane 2: positive control (T. evansi); Lanes 4–8 and 10–11: positives for T. evansi (488 bp); Lanes 3 and 9: negative samples. (TIF 86 kb) [file 13071_2017_2117_MOESM2_ESM.tif]

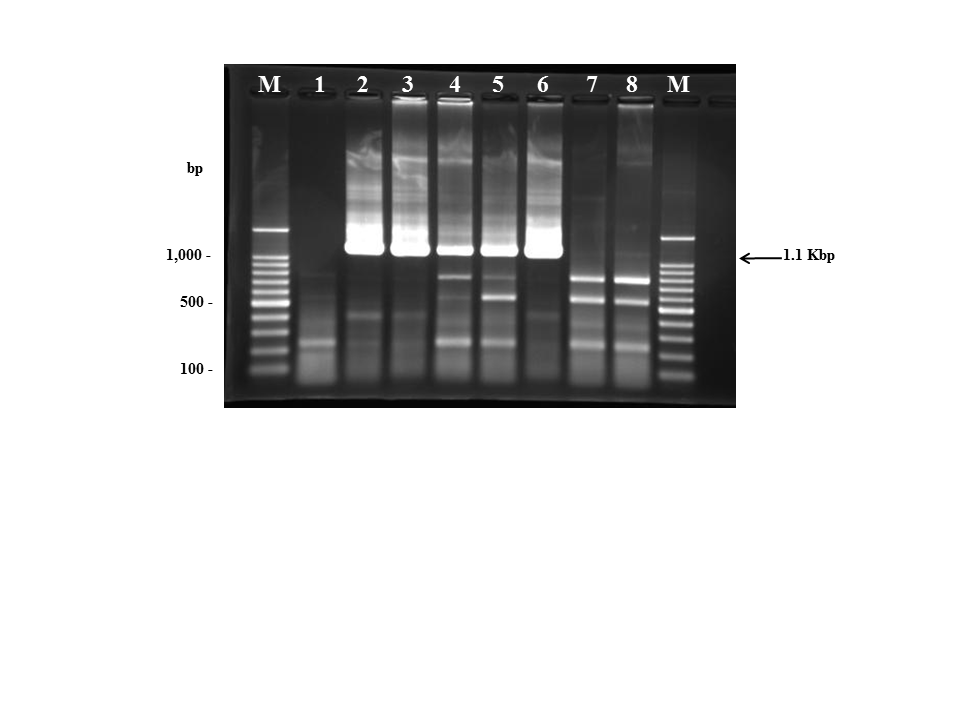

Supplement: Supplementary file 3 — Agarose gel electrophoresis (1.5%) with ethidium bromide staining of field isolates of T. evansi. DNA was amplified with an ITS-PCR (IR1 and IR2 primers). Lane M: 100-bp marker; Lane 1: negative control; Lane 2: positive control (T. evansi); Lanes 3–6: positives for T. evansi (1.1 kbp); Lanes 7 and 8: negative samples. The extra-bands were non-specific. (TIF 144 kb) [file 13071_2017_2117_MOESM3_ESM.tif]

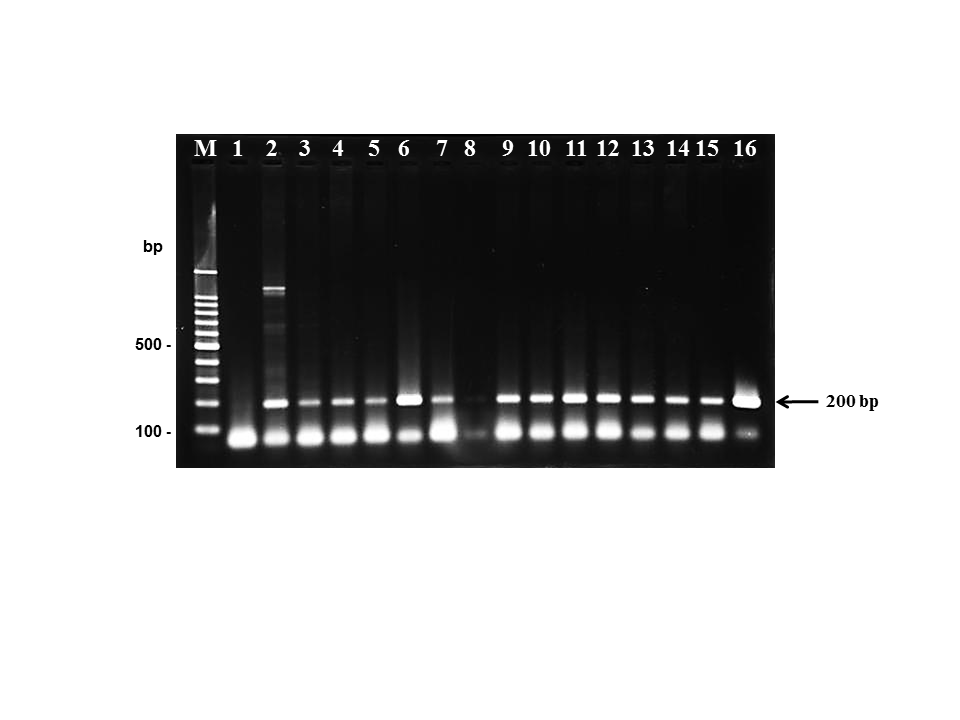

Supplement: Supplementary file 4 — Agarose gel electrophoresis (2%) with ethidium bromide staining of field isolates of T. vivax. DNA was amplified with a TviCatL-PCR (DTO155 and TviCatL1 primers). Lane M: 100-bp marker; Lane 1: negative control; Lanes 2–7 and 9–15: positives for T. vivax (200 bp); Lane 16: positive control (T. vivax); Lane 8: negative sample. The extra-bands were non-specific. (TIF 122 kb) [file 13071_2017_2117_MOESM4_ESM.tif]
